# Automated vetting of radiology referrals: exploring natural language processing and traditional machine learning approaches

**DOI:** 10.1186/s13244-022-01267-8

**Published:** 2022-08-04

**Authors:** Jaka Potočnik, Edel Thomas, Ronan Killeen, Shane Foley, Aonghus Lawlor, John Stowe

**Affiliations:** 1 University College Dublin School of Medicine, Dublin, Ireland; 2 University College Dublin School of Computer Science, Dublin, Ireland

**Keywords:** Machine learning, Natural language processing, Justification audit, Radiology referral, Clinical decision support

## Abstract

**Background:**

With a significant increase in utilisation of computed tomography (CT), inappropriate imaging is a significant concern. Manual justification audits of radiology referrals are time-consuming and require financial resources. We aimed to retrospectively audit justification of brain CT referrals by applying natural language processing and traditional machine learning (ML) techniques to predict their justification based on the audit outcomes.

**Methods:**

Two human experts retrospectively analysed justification of 375 adult brain CT referrals performed in a tertiary referral hospital during the 2019 calendar year, using a cloud-based platform for structured referring. Cohen’s kappa was computed to measure inter-rater reliability. Referrals were represented as bag-of-words (BOW) and term frequency-inverse document frequency models. Text preprocessing techniques, including custom stop words (CSW) and spell correction (SC), were applied to the referral text. Logistic regression, random forest, and support vector machines (SVM) were used to predict the justification of referrals. A test set (300/75) was used to compute weighted accuracy, sensitivity, specificity, and the area under the curve (AUC).

**Results:**

In total, 253 (67.5%) examinations were deemed justified, 75 (20.0%) as unjustified, and 47 (12.5%) as maybe justified. The agreement between the annotators was strong (*κ* = 0.835). The BOW + CSW + SC + SVM outperformed other binary models with a weighted accuracy of 92%, a sensitivity of 91%, a specificity of 93%, and an AUC of 0.948.

**Conclusions:**

Traditional ML models can accurately predict justification of unstructured brain CT referrals. This offers potential for automated justification analysis of CT referrals in clinical departments.

## Key points

• Unjustified exposure to CT scans increases lifetime radiation risk of stochastic effects.
• Along with budgetary needs, auditing justification of radiology referrals is time-consuming.
• CDS ensures audit consistency and less discrepancies between human experts.
• ML algorithms used the clinical indications section of radiology referrals for classification.
• ML algorithms can accurately predict justification of brain CT referrals.

## Background

Computed tomography (CT) scans are associated with relatively high radiation doses, and as a result, patients are potentially at greater lifetime risk of developing a radiation-induced cancer [1]. Many patients undergo multiple CT scans; therefore, their cumulative risk of developing a radiation-induced cancer is significantly higher [2]. Since 2009, the number of CT examinations carried out in Ireland has almost doubled [3]. A similar trend is seen in the UK where the CT scan frequency between 2012 [4] and 2019 [5] increased by approximately 74%, while a 20% increase occurred in the USA in the 2006–2016 period [6]. Increasing CT frequency poses additional population dose burden, as CT is the largest dose contributor [7]. National audits from Northern Ireland [8], Sweden [9], and Luxembourg [10], report 6%, 19%, and 39% of unjustified CT examinations, respectively. Furthermore, individual local audits within Europe indicate poor justification practice with the rate of unjustified CT examinations between 7 and 30% [11–14]. To improve justification of CT examinations in the European Union, the European Commission recently funded a 3-year project to co-ordinate audits of justification of CT examinations and to develop a common methodology for auditing justification of CT referrals [15]. Avoiding unnecessary exposure to ionising radiation is the primary evidence-based intervention that reduces cancer risk [1, 2]. The information contained in radiology referrals is manually used during justification, for assessing their compliance with clinical imaging guidelines which aim to improve patient care through evidence-based recommendations of radiology resources. Clinical decision support (CDS) systems are not yet commonly used in radiology, and the unstructured form of electronic radiology referrals requires preprocessing.

Natural language processing (NLP) refers to a set of techniques for preparing text data and converting it into a structured form suitable for subsequent machine learning [16, 17]. It is common in NLP and machine learning research to represent natural text as a term frequency-inverse document frequency (TF-IDF) model, or a bag-of-words (BOW) model where feature values correspond to term frequency in a document. The BOW model converts each text document into an n-dimensional vector where n is the number of unique terms or vocabulary size, and the vector values are the occurrence counts or term frequency (TF) of those terms. It is usual to correct the counts of common words with an inverse document frequency (IDF) term, and the combination of both, TF-IDF, is a reasonable model for representing the importance of words in a text corpus [18]. Machine learning is an automated process of detecting underlying patterns within data. In predictive data analytics, classification models that identify the relationship between a set of descriptive features and a target feature on retrospectively collected data are built. When the ground truth labels are known, models are in the category of supervised machine learning [19]. Although traditional machine learning (ML) techniques are often surpassed by deep learning methods, they do offer high performance on smaller textual datasets with great potential for a real-time CDS [20–22]. Past research in the field of radiological NLP has mainly focused on the radiological report [16, 17, 23, 24]. There seems to be a lack of research on the use of radiological NLP in referring practices; hence, our study aims to explore the potential of CDS in radiology by applying NLP-based models to the most common CT referral nationally—brain CT [25] to predict justification according to audit outcomes. The optimal text preprocessing pipeline for unstructured clinical text in radiology referrals is not known. Different text preprocessing pipelines for unstructured brain CT referrals were developed. Subsequently, ML approaches for classification of CT referrals from a single tertiary referral hospital according to the vetting outcomes were evaluated.

## Methods

This retrospective research underwent a local data protection impact assessment and was exempt from full ethics review. The research was granted an ethics exemption (REERN: LS E-21-82-Potocnik-Stowe). All anonymised data were encrypted and stored on a university cloud storage in a comma-separated values format. Data science tasks were performed using Python (version 3.7.11).

### Data collection

The study included all adult brain CT examinations obtained from a tertiary referral hospital based in Dublin, Ireland, in a single calendar year (2019). Anonymised referrals were extracted from the radiology information system (Carestream Vue, Rochester, NY, USA). These included outpatient, inpatient, and emergency referrals with different numbers of referrals in each of these categories. For each referral, patient gender, patient age, scan priority level, and unstructured clinical indications were recorded. The referrals at this clinical site were handwritten and thus were manually transcribed in an encrypted Excel spreadsheet without alterations being made to the grammatical structure of the unstructured text.

### Data annotation

**Fig. 1:**
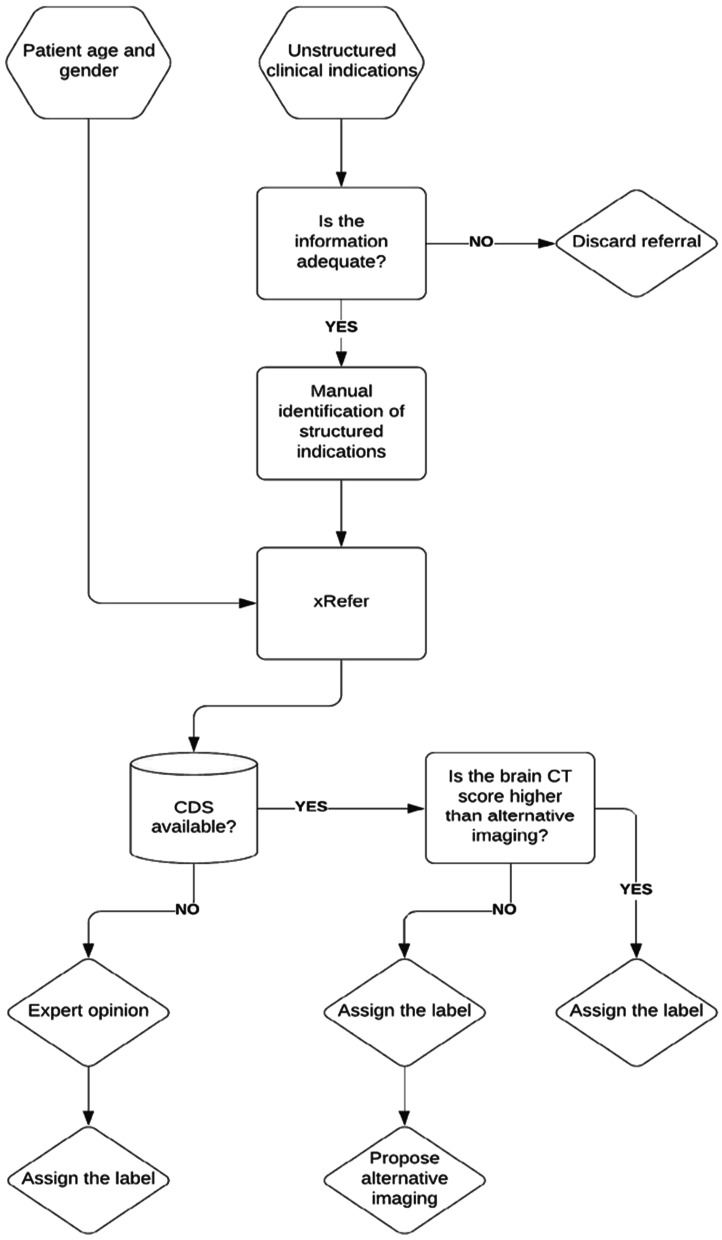
Semi-automated vetting pipeline for brain CT referrals

The dataset was manually inspected and independently annotated by a consultant neuroradiologist (10 years of experience) and a radiographer (5 years of experience). Both annotators used xRefer (xWave, Dublin, Ireland), a cloud-based CDS tool for structured referring (version 1.12.1-uat) [26] based on referral criteria for imaging developed by the European Society of Radiology [27] in cooperation with the American College of Radiology (ACR) [28], to audit justification of the referrals. Given the patient baseline characteristics, xRefer outputs justification scores associated with imaging modalities and examination types for a selected structured clinical indication. Considering the ACR scoring system, each referral could fall into one of the three categories: justified (score: 7–9), maybe justified (score: 4–6), and unjustified (score: 0–3). This process of semi-automated vetting with xRefer comprised of four steps. Firstly, 79 (17.4%) referrals were discarded due to inadequate clinical information. Information was deemed inadequate if a structured clinical indication could not be extracted from the unstructured clinical text due to brief wording or excessive variations in interpretation. To obtain justification scores for each referral, patient gender and age were also included. Secondly, upon manual interpretation of the unstructured clinical text and identifying one or more structured clinical indications, the identified structured indication was found in the platform’s database via its search engine. The structured indication of interest was confirmed to produce justification scores. Thirdly, if the identified structured indication was not found in the xRefer’s database, or the CDS scores were unavailable for a selected structured indication, such referrals were labelled based on the consultant’s opinion. When two or more structured indications were identified on a single referral, only the highest scoring indication was taken into consideration for justification purposes. If applicable, an alternate, more appropriate imaging method was proposed. Lastly, discrepancies in labels obtained using xRefer’s CDS system were addressed individually by both annotators and a final decision on a label was made by consensus. The vetting pipeline is summarised in Fig. 1. The annotated referrals were grouped into five categories depending on adequacy of information and availability of CDS with xRefer: (1) all referrals, (2) referrals with adequate information, (3) referrals with adequate information and with CDS scores, (4) referrals with adequate information and without CDS scores, and (5) referrals with adequate information and no matching structured indication. Cohen’s kappa (*κ*) was computed for each group to determine the inter-rater agreement.

### Classification task

Considering the categorisation of referrals in the national audits across Europe [8–10], and due to the nature of unstructured writing and a non-representative multiclass dataset, the referrals of questionable justification have been considered as unjustified. The new dataset served as an experimental dataset for binary classification.

### Text preprocessing

The unstructured clinical indications within each referral represent features, as they contain the most information that contributes towards examination justification. The text was preprocessed using Python’s Natural Language Toolkit (NLTK, version 3.6.5) library. All sentences were converted to lowercase and tokenised with the NLTK WordTokenizer. A default stop word list, containing NLTK’s stop words and punctuation marks, was applied to filter redundant tokens. Furthermore, custom stop words were identified by manually inspecting each referral and a token list with associated counts. To reduce noise, unigrams with document frequency equal to one were ignored. Misspelled tokens were corrected with Pyspellchecker (version 0.6.2) incorporating a custom medical dictionary containing 442 clinical terms that were identified in our vocabulary. Rare clinical terms were replaced by one of their synonyms. Features were represented as a BoW model with CountVectorizer and a TF-IDF model with TfidfVectorizer from the Scikit-learn library.

### Model evaluation

The experimental dataset for binary classification was randomly divided (80/20) into a training set and a test set. The training set contained 300 (80% of the whole dataset) referrals that were used to train the prediction models with default hyperparameter settings and balanced class weights to penalise misclassification errors for the minority class. The test set contained 75 (20% of the whole dataset) referrals that were used to demonstrate classifier performance on the new, previously unseen referrals. Weighted accuracy score, sensitivity, specificity, and area under the curve (AUC) were computed for each classifier. Scikit-learn (sklearn, version 1.0.1) was used to evaluate combinations of different text preprocessing techniques and classifiers.

## Results

### Justification audit

Table 1 demonstrates the kappa scores, as well as the number of referrals belonging to each group associated with information adequacy and availability of CDS with xRefer. There was a significant difference (*p* < 0.01) in the inter-rater agreement between referrals that fall under CDS and those without CDS.

**Table 1:** Referral grouping and associated inter-rater agreement between the two human experts

Group	Frequency	*κ* score
All referrals	454	0.770
Referrals with adequate information	375	0.835
Referrals with adequate information and with CDS scores	327	0.874
Referrals with adequate information and without CDS scores	29	0.408
Referrals with adequate information and no matching structured indication	19	0.506

In total, 253 (67.5%) examinations were considered justified, 75 (20.0%) unjustified, and 47 (12.5%) maybe justified. In total, 96 (25.6%) CT scans could have been replaced by magnetic resonance imaging (MRI). Symptoms of dizziness, syncope/fainting, vision changes, chronic headache, headache in cancer patients, maxillofacial headache, sub-dural haemorrhage, tinnitus, and dementia may indicate a need for an MRI. All oncology referrals (6) were inappropriate, as they required a brain CT scan for non-small cell lung cancer (NSCLC) or prostate cancer staging post-treatment cycle. iGuide imaging guidelines suggest that a brain CT is justified in cases of NSCLC staging only if MRI is contraindicated, and neurological symptoms are present; however, there was no such information provided by the referrer. The absence of CDS scores for prostate cancer diagnosis and staging meant that such requests were determined by consultant opinion which concurred with iGuide recommendations for NSCLC imaging. Five (1.3%) patients needed a facial bone CT scan, rather than a brain CT scan: four patients had sustained facial trauma without accompanying suspicion of intracerebral and/or intracranial complication. One patient exhibited symptoms of cerebrospinal fluid leak post-lumbar decompression, and CT cisternography was more appropriate. Another patient with vocal cord paralysis for 12 weeks, should have had a brain and neck CT instead of brain CT according to iGuide. Three (0.8%) of the 375 CT examinations could have been replaced by ultrasound (US). Patients experiencing nausea and/or vomiting without known head injury should have undergone abdominal US scan initially. All indications, except for vision changes, specified on referrals of questionable justification indicate a need for a head MRI. Symptoms of vision changes indicate that an MRI of the orbits is the investigation of choice.

### ML algorithms

Table 2 shows classifier performance in combination with different text preprocessing pipelines on the binary dataset. A combination of BOW representation and SVM outperformed the rest of the models. The best performing models were BOW + DSW + SVM and BOW + CSW + SC + SVM with superior AUC of 0.942 and 0.948, respectively.

**Table 2:** Binary classifier evaluation metrics on test set

Model	Weighted accuracy (%)	Sensitivity (%)	Specificity (%)	AUC
BOW + DSW + LR	88.3	86.7	90.0	0.925
BOW + DSW + SVM	92.8	88.9	96.7	0.942
BOW + DSW + RF	88.3	86.7	86.7	0.930
TF-IDF + DSW + LR	87.2	84.4	90.0	0.923
TF-IDF + DSW + SVM	86.1	88.9	83.3	0.923
TF-IDF + DSW + RF	85.0	86.7	86.7	0.931
BOW + CSW + LR	87.2	84.4	90.0	0.915
BOW + CSW + SVM	88.9	84.4	93.3	0.932
BOW + CSW + RF	85.6	84.4	86.7	0.910
TF-IDF + CSW + LR	85.0	80.0	90.0	0.917
TF-IDF + CSW + SVM	85.0	80.0	90.0	0.926
TF-IDF + CSW + RF	85.6	84.4	86.7	0.910
BOW + CSW + SC + LR	87.2	84.4	90.0	0.932
BOW + CSW + SC + SVM	92.2	91.1	93.3	0.948
BOW + CSW + SC + RF	87.2	84.4	90.0	0.911

*BOW* bag-of-words, *DSW* default stop words, *CSW* custom stop words, *LR* logistic regression, *RF* random forest, *SC* spell checker, *SVM* support vector machine

## Discussion

Based on the Kappa scores, our approach to auditing justification of radiology referrals with xRefer shows better consistency and less discrepancies between the two annotators, compared to auditing radiology referrals without CDS, regardless of the gap in clinical expertise and knowledge. Since the performance of a prediction model depends significantly on the quality of annotations, the audit findings suggest that xRefer can be used to conduct a retrospective, semi-automated, evidence-based justification analysis of CT referrals, although the platform has been primarily created for structured referring.

The rate of unjustified examinations (20.0%) and those of questionable justification (12.5%) indicate that clinical imaging guidelines have not been adapted and implemented within clinical practice adequately. Several factors, such as lack of awareness of clinical imaging guidelines, time, patient pressure, along with privatisation of healthcare and prioritising economic gain [29], may influence justification decisions. This correlates with our findings such as clinical indications of ‘investigations’ and ‘dizziness/Lt tinnitus’ specified on two unjustified referrals.

Regular audits of justification are necessary to ensure better implementation of clinical imaging guidelines, prevent unnecessary patient radiation dose, limit wasteful use of resources, and improve patient care. Retrospective audits are costly, time-consuming, and often not feasible. In this study, an iGuide interpreter was developed for automated justification analysis of clinical indications specified on brain CT referrals. The NLP model can automatically predict justification of brain CT referrals, consequently offering a potential for automated clinical text analysis that could be used as a CDS tool to assist referrers and practitioners with justifying brain CT referrals, retrospectively and in real time. This would allow more frequent, cost-free justification audits, and better implementation of imaging referral guidelines within clinical practice to ensure appropriate justification. To our knowledge, this is the first study evaluating ML methods in predicting justification of radiology referrals. Similar work has been done where NLP and ML approaches were applied to the conclusions section of CT reports to automatically predict downstream radiology resource utilisation in patients undergoing surveillance for hepatocellular carcinoma [21]. The study demonstrates that even with minimal text preprocessing, a linear model can achieve an accuracy of 92.2%. In contrast, our study involves CT referrals and more data preprocessing which may or may not improve classifier performance. Our linear model achieved a weighted accuracy of 92.8% with minimal data preprocessing and 92.2% after introducing custom stop words and spell correction with Pyspellchecker, but the second model had a higher sensitivity and AUC. There are three important data preprocessing elements that affect classifier performance:

1. *Data representation*—as a group, BOW models tend to outperform TF-IDF models. We note that terms associated with unjustified referrals also occur in justified referrals and vice versa (Table 3). In some cases, justified and unjustified terms occur together within a single referral. All of this neutralises the effect of a TF-IDF approach.
2. A classifier might deem clinically irrelevant terms important for classification of CT referrals and when these are ignored, the classifier’s performance may decrease. This also challenges machine learning ethics with the question of including clinically irrelevant terms to achieve better accuracy.
3. *Spelling correction* We corrected misspelled clinical terms with Pyspellchecker. The spell-checking algorithm uses Levenshtein distance and is limited to an edit distance of two; therefore, the algorithm is unable to correct misspelled terms if more than two permutations are needed. In some instances, misspelled terms were wrongly corrected, so the algorithm’s output was either ignored due to producing noise, or accepted as a spurious term. For example, one referral reads: “prostate cancer-re staging” and the algorithm corrected the sentence into “prostate sancerre staging”. On the other hand, a misspelled “eposide” was replaced with “epoxide” instead of “episode”. We note cases where the algorithm did not make any corrections as the authentic word was misspelled, given a context and yet, grammatically correct: “head ache” should have been combined into a single word “headache”.

**Table 3:** Document frequency of terms associated with justified and unjustified referrals

Term	Document frequency associated with a positive class	Document frequency associated with a negative class
Fall	72	8
Headstrike	8	3
Headaches (chronic)	9	18

Implementing a custom stop word list and spell-checking algorithm may improve classifier performance. 43.5% of referrals in our training set were unjustified. Our test set contained 45 justified and 30 unjustified referrals. This is a mild degree of imbalance. SVM models tend to be less sensitive to class imbalance as they try defining a hyperplane that separates examples belonging to each class in a high-dimensional space, thereby achieving higher accuracy compared to other models which strive towards minimising the error rate [30].

In terms of false predictions, BOW + CSW + SC + SVM made two false-positive and four false-negative predictions. An analysis of false positives revealed that certain terms, such as “fall” and “headstrike”, downgraded the model’s performance because they are associated with both classes, more commonly with a positive class. When referrers provide clinical indications, a rationale needs to be included. Relatively often, the rationale is inappropriate or absent. For example:

1. Fall, headstrike, takes aspirin.
2. CT brain—fall headstrike uses NOAC.
3. Fall with headstrike—bruise L forehead.

A rationale behind the first two referrals justifies a brain CT scan as there is a high suspicion of intracerebral haemorrhage due to anticoagulant therapy. In contrary, a bruised forehead alone is not sufficient to justify a brain CT scan after falling; however, the third referral has been misclassified as a false positive.

In terms of false negatives, CT referrals include terms associated with both categories, but more frequently appear in unjustified referrals.

1. Ongoing headaches.
2. Fall down stairs HI Headaches.
3. Ongoing headaches over 12/12. The headaches tend to radiate from the back of his neck. ?Aetiology of headaches. The DDX include tension-type headaches, but the history of episodic blurred vision and ataxia make the diagnosis more difficult.

The first referral reads as a chronic headache and without any further rationale it was deemed as unjustified. The second referral raises a concern over a post-traumatic headache associated with a traumatic brain injury [28] which justifies a brain CT scan. The third referral, misclassified as a false negative, contains focal neurological symptoms in conjunction with a chronic headache; therefore, imaging with CT is indicated.

Our study had certain limitations. First, the benefits of CDS for auditing justification of CT referrals with xRefer were not demonstrated to a full extent, as the comparison groups of CDS and non-CDS referrals are significantly different in size. Second, our data were sourced from a single clinical site; therefore, it is premature to make assumptions that NLP-based models can generalise to unseen datasets. Third, as our dataset was small and imbalanced, referrals of questionable justification were considered as unjustified. While this is acceptable [8–10], it is desirable for an iGuide interpreter to classify referrals into one of the three possible classes as per iGuide categorisation. Fourth, trained models do not take patient gender, age, urgency level, and prior imaging into account. Lastly, the results of this study may not reflect true prediction capabilities as hyperparameters were not optimised. Nevertheless, the models achieved high accuracy regardless of the limitations of the dataset.

## Conclusions

Unjustified exposure to CT scans is a global problem that increases lifetime radiation risk of stochastic effects, resource wastage, and CT waiting lists. Our NLP-based models can accurately predict justification of brain CT referrals and, as a result, offer potential for automation of this time-consuming, often costly process. More NLP research is needed to address justification of other types of CT scans and different imaging modalities to explore the potential of automated justification analysis in clinical departments.

## Data Availability

The datasets generated during and/or analysed during the current study are available from the corresponding author on reasonable request.

## Abbreviations

ACR: American College of Radiology
AUC: Area under the curve
BOW: Bag-of-words
CDS: Clinical decision support
CSW: Custom stop words
CT: Computed tomography
DSW: Default stop words
LR: Logistic regression
ML: Traditional machine learning
MRI: Magnetic resonance imaging
NLP: Natural language processing
NLTK: Natural language toolkit
NSCLC: Non-small cell lung cancer
RF: Random forest
SC: Spell checker
SVM: Support vector machine
TF-IDF: Term frequency-inverse document frequency
US: Ultrasound
